# Optical–Electronic Skin Based on Tea Polyphenol for Dual Signal Wearable Sensing

**DOI:** 10.3390/bios15050281

**Published:** 2025-04-29

**Authors:** Jia-Li Xu, Guangyao Zhao, Jiachen Wang, An Tang, Jun-Tao Liu, Zhijie Zhu, Qiang Zhang, Yu Tian

**Affiliations:** 1 School of Chemistry and Chemical Engineering, Key Laboratory of Surface & Interface Science of Polymer Materials of Zhejiang Province, Zhejiang Sci-Tech University, Hangzhou 310018, China; 2023221002065@mails.zstu.edu.cn (J.-L.X.); 202230107398@mails.zstu.edu.cn (A.T.); 2023211001026@mails.zstu.edu.cn (J.-T.L.); 2 Department of Biomedical Engineering, City University of Hong Kong, Kowloon, Hong Kong 999077, China; guangzhao7-c@my.cityu.edu.hk (G.Z.); jiacwang7-c@my.cityu.edu.hk (J.W.); 3 Jiangsu Advanced Textile Engineering Technology Center, Jiangsu College of Engineering and Technology, Nantong 226007, China; zhuzj@jcet.edu.cn; 4 Institute of Digital Medicine, City University of Hong Kong, Kowloon, Hong Kong 999077, China

**Keywords:** optical–electronic skin, structural color, dual-signal output, dynamic boronate ester bonds

## Abstract

The rapid development of smart electronic skin has led researchers to design a variety of flexible and stretchable devices that can be used to monitor physiological and environmental signals. In this work, we successfully demonstrate a color-adjustable and conductive wearable optical–electronic skin (OE-skin) based on photonic crystal hydrogel that is capable of delivering both optical and electrical signal responses synchronously. The OE-skin is fabricated by incorporating a structural colored layer, composed of periodically aligned magnetic nanoparticles, into a polyacrylamide hydrogel matrix that contains tea polyphenols and borax. The dynamic boronate ester bonds formed between borax and the catechol groups of tea polyphenols are able to enhance the mechanical properties of the OE-skin, while also conferring excellent electrical conductivity, high sensitivity, and a rapid electrical response. Additionally, the tea polyphenols, which are natural active compounds derived from tea, possess diverse bioactive properties, thereby endowing the OE-skin with excellent antibacterial and biocompatibility characteristics. In addition, the developed electronic skin successfully demonstrates its capability in synergistic electronic and optical sensing during human motion monitoring, indicating broad application prospects in the field of smart wearable sensors.

## 1. Introduction

Inspired by the ability of chameleons and other organisms to sense external stimuli and adaptively respond to a complex environment, researchers have developed a large number of artificial electronic skins (E-skins) [1,2]. An E-skin mimics human skin by sensing external environmental factors, including pressure [3], temperature [4,5,6], humidity [7], and others, and converting this information into electrical signals. This functionality is crucial for applications in intelligent wearable sensors, human motion monitoring, and intelligent robotics, among other domains. However, current E-skins typically exhibit a single pattern of electrical signal output and lack chameleon-like visual feedback capabilities.

To address the limitation of single-mode sensing, researchers have incorporated color elements, such as pigments [8] and fluorescent materials [9,10,11], into E-skin, thereby developing optical/electrical skin (OE-skin). However, the inherent disadvantages of these color elements, such as easy photobleaching, low contrast, and slow reaction, affect the stability of the sensor signal transmission [12]. A photonic crystal with periodic structure has the advantages of stable structural color, visible response, and high contrast [13]. More importantly, the lattice constant of the photonic crystal is instantaneously adjusted in response to the matrix deformation, enabling the precise tracking of external stimuli through structural color changes [14]. The optical and electrical responsiveness of intelligent skin can be realized by embedding photonic crystal structure into the conductive matrix to enable the dual-signal transmission synchronously [15,16,17]. To achieve conductivity and enhance the electrical signal transmission, various fillers, including carbon materials [18,19,20], ionic liquids [21,22], and noble metal nanoparticles [23], are commonly incorporated into the supporting OE-skin. For example, Wang et al. successfully incorporated a carbon nanotube–polydopamine composite into an inverse photonic crystal polyurethane (PU) film, thereby achieving an optical/electrical dual-signal response for highly sensitive human-motion sensing [24]. However, the poor interfacial compatibility of fillers and polymeric matrices often lead to compromised mechanical properties. Other strategies to improve conductivity include directly incorporating conductive polymers, such as polyaniline and polypyrrole, into the hydrogel matrix. Ye et al. proposed a stretchable conductive OE-skin that can provide mechanical compliance and high conductivity by polymerizing pyrrole on the surface of a PU film [25]. However, further efforts are essential to enhance the mechanical properties, specifically stretchability and durability, while simultaneously taking the response rate, biocompatibility, and antibacterial capabilities into consideration, as these factors are essential for ensuring the performance and long-term reliability of OE-skin.

Tea polyphenol (TP), a type of plant polyphenols, exhibits favorable antibacterial, anti-inflammatory, biocompatible, and biodegradable properties [26,27]. The abundant phenolic hydroxy groups within TP can form hydrogen bonds with polar groups of polymer chains in the hydrogel matrix, thereby enhancing the mechanical strength [28,29]. Moreover, TP can establish strong interactions with diverse surfaces, making it an ideal candidate for the preparation of self-adhesive hydrogels [30]. Notably, the catechol groups in TP can dynamically form boronate ester bonds (TP-B) with borate ions released from borax ionization, creating a stable, biocompatible interface within the hydrogel matrix while improving its conductive properties [31].

In this article, we present a tea polyphenol/borax photonic crystal (TPC) OE-skin designed for the dual-signal sensing of human motions. This TPC OE-skin was fabricated by incorporating Fe_3_O_4_@C nanoparticles, which function as photonic structural units, into a polyacrylamide (PAm) hydrogel matrix doped with tea polyphenols and borax. The aligned magnetic Fe_3_O_4_@C nanoparticles embedded within the hydrogel matrix enable the generation of reflective light, producing structural colors that can be directly observed by the naked eyes or recorded with a camera. The dynamic boronate ester bonds formed between tea polyphenols and borax endow the TPC OE-skin with high conductivity and excellent mechanical stability, ensuring reliable electrical signal output. The mechanical deformation of the TPC OE-skin not only induces the relative resistance change but also enables the simultaneous adjustment of the lattice constant in the magnetic Fe_3_O_4_@C nanoparticle arrays, thereby realizing the modulation of structural colors. These functionalities allow the OE-skin to exhibit synergistic electrical and optical dual-signal feedback in response to deformation stimuli. Furthermore, the incorporation of tea polyphenols enhances the adhesion, antibacterial properties, and biocompatibility of the OE-skin, highlighting its potential as an effective biological interface for wearable device applications. Our results demonstrate that the TPC OE-skin enables synergetic optical and electrical signal sensing during human motion monitoring, underscoring its significant application value in fields such as human health monitoring and intelligent wearable devices.

## 2. Experimental Section

### 2.1. Chemicals and Materials

Ferrocene (98%), ethylene glycol (EG > 99%), ethanol (99.5%), methanol (99.5%), tea polyphenol (TP, EGCG, 98%), and sodium tetraborate decahydrate (99.5%) were purchased from Aladdin (Shanghai, China); Acrylamide (Am, 99%), N, N′-methylene-bis-acrylamide (MBA, >99%), and 2-Hydroxy-4′-(2-hydroxyethoxy)-2-methylpropiophenone (photoinitiator 2959, 98%) were purchased from Macklin (Shanghai, China). Hydrogen peroxide (H_2_O_2_, 30 wt.%) was supplied by Lingfeng Co., Ltd. (Shanghai, China). Acetone was supplied by Shuanglin Chemical Co., Ltd. (Hangzhou, China). All the chemicals were used as received.

### 2.2. Preparation of Fe_3_O_4_@C Nanoparticles

A mass of 0.54 g of ferrocene was dissolved in 42.5 g of acetone in a brown bottle, and the mixture was subjected to ultrasonication for 30 min to yield a uniform yellow solution. Subsequently, varying amounts of H_2_O_2_ (1.2–1.45 mL) were added dropwise to the solution under continuous stirring, followed by an additional 3 h of stirring. The resulting precursor was then transferred to a 100 mL hydrothermal reactor and heated at 220 °C for 72 h. After the reaction, the product was collected using a magnet and washed three times with anhydrous ethanol to remove any unreacted impurities. Finally, the product was dried at 60 °C in an oven. For further utilization, 0.04 g of Fe_3_O_4_@C nanoparticles were dispersed in 10 mL of EG and sonicated to form a stable Fe_3_O_4_@C/EG dispersion.

### 2.3. Preparation of TPC OE-Skins

Am, 2959, MBA, TP, and borax were sequentially added to 1.4 mL of EG and thoroughly mixed via ultrasonication to form a homogeneous solution. The weight ratio of TP to borax was maintained at 4:1. Detailed formulations of the TPC OE-skins with varying amounts of TP are provided in Table S1. This solution was subsequently combined with 1 mL of Fe_3_O_4_@C/EG solution and further dispersed by ultrasonication. The resulting mixture was dropwise added onto a polytetrafluoroethylene (PTFE) mold covered with a PET film (4 cm × 2 cm). The mixture was then exposed to ultraviolet light at 365 nm for five minutes to initiate the polymerization process, while a magnetic field was applied at the bottom of the mold to achieve the formation of the TPC OE-skins. PC hydrogels were fabricated using the same procedure but without TP or borax.

### 2.4. Characterization

The morphology of the Fe_3_O_4_@C nanoparticles was characterized using transmission electron microscopy (TEM, JEM-2100, JEOL, Japan). The crystal structure of the Fe_3_O_4_@C nanoparticles was analyzed via X-ray diffraction (XRD) using a Bruker AXS D8 Discover diffractometer. Magnetic properties were evaluated using a vibrating sample magnetometer (VSM, 7404, LakeShore, Carson, CA, USA). The reflectance spectra of the TPC OE-skin were measured with an optical microscope integrated with a fiber spectrometer (USB 4000, Ocean Optics, USA). The microstructure of the TPC OE-skin was examined by scanning electron microscopy (SEM, S-4800, Hitachi, Tokyo, Japan) after freeze-drying. Mechanical properties were assessed using a universal testing machine. Rheological behavior was investigated using a rotational rheometer (Kinexus Lab+, NETZSCH, Germany).

### 2.5. Biocompatibility Testing

L-929 cells were used to evaluate the biocompatibility of the prepared PC hydrogel samples and TPC OE-skin samples. Cultivate L-929 cells in Dulbecco’s Modified Eagle’s Medium (DMEM, Gibico, Shanghai, China), containing 10% fetal bovine serum (FBS, Gibico, China) and 1% penicillin/streptomycin (PS, Gibico, Shanghai, China). L-929 cells were seeded in a 24-well plate and incubate for 24 h. The samples were mixed with culture directly for 24 h to test the survival rate. Live and dead staining (Beyotime, Shanghai, China) was used to evaluate the cell viability according to the manufacturer’s instructions.

### 2.6. Antimicrobial Testing

The surface antimicrobial properties of the negative control (without hydrogel addition), PC hydrogel (without TP-B), and TPC OE-skin were evaluated against representative strains of *Escherichia coli* (*E. coli*) and *Staphylococcus aureus* (*S. aureus*). Each sample was aseptically transferred to a sterile tube, followed by the addition of 100 μL of bacterial suspension (3.5 × 10^4^ CFU mL^−1^) onto the surface. The samples were incubated at 37 °C for 6 h. After incubation, the bacterial suspensions were serially diluted 10-fold, and 100 μL aliquots were plated onto agar plates, which were then incubated at 37 °C for 18 h. Colony-forming units (CFU) were counted to determine the antimicrobial efficacy of TPC OE-skin. All experiments were conducted in triplicate. The formula for calculating the logarithmic reduction is as follows:

(1) $$log\;reduction=\log\left(survivor\;cell\;count\;of\;negative\;control\right)\;\;\;\;\;\;-\log(survivor\;count\;cell\;of\;samples)$$

### 2.7. Electrical Testing and Human Motion Monitoring

The conductivity (σ) of TPC OE-skin is analyzed by LCD digital bridge (TH2830, Changzhou Tonghui Electronics Co., Ltd., Changzhou, China). *σ* is calculated as follows:

(2) σ=l/(R×S)

where *l* is the distance between the electrodes, *R* is the measured resistance, and *S* is the cross-sectional area of the OE-skin. The electrical properties of TPC OE-skin were evaluated under different strains. The relative resistance change of the OE-skin was calculated using the following formula:

(3) $$\Delta R/R_{0}=(R-R_{0})/R_{0}\times 100\%$$

where *R*_0_ is the resistance in the initial state and *R* is the real-time resistance under a specific strain. To evaluate the sensitivity of the relative resistance change to strain, the gauge factor (GF) of the OE-skin was assessed using the following formula:

(4) $$GF=(\Delta R/R_{0})/\varepsilon$$

where ε is the strain change.

## 3. Results and Discussion

### 3.1. Fabrication of TPC OE-Skins

**Figure 1:**
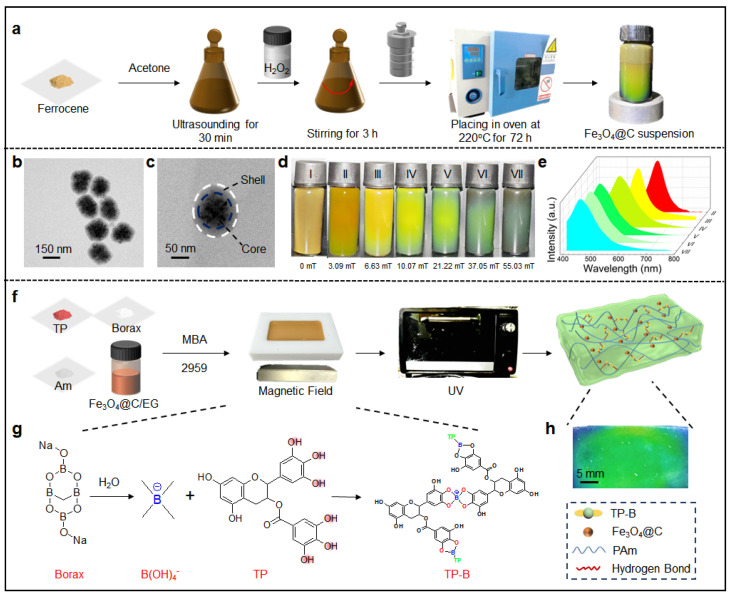
(**a**) The synthesis procedure of Fe_3_O_4_@C nanoparticles; (**b**,**c**) TEM images of the Fe_3_O_4_@C nanoparticles; (**d**) Digital photographs showing the color changes of Fe_3_O_4_@C/EG dispersion under varying magnetic fields; (**e**) Reflection spectra of Fe_3_O_4_@C/EG dispersion under different magnetic fields; (**f**) Schematic representation of the preparation process for TPC OE-skin; (**g**) Schematic diagram depicting the dynamic formation of boronate ester bonds; (**h**) Digital photographs of TPC OE-skin.

Fe_3_O_4_@C nanoparticles were synthesized via a hydrothermal method by introducing hydrogen peroxide into the precursor solution of ferrocene and acetone. The Fe_3_O_4_@C nanoparticle solution exhibited vivid structural colors in a magnetic field (Figure 1a). TEM images revealed that the Fe_3_O_4_@C nanoparticles are spherical with an average diameter of 150 nm (Figure 1b), and their core–shell structure is clearly visible. Specifically, the Fe_3_O_4_ nanocrystals form clustered cores, while carbon constitutes the shell layers (Figure 1c). The saturation magnetization of these nanoparticles is 40.08 emu/g, indicating strong superparamagnetic properties (Figure S1). XRD analysis confirmed that the diffraction peaks correspond to Fe_3_O_4_@C, whereas the amorphous carbon shells do not exhibit any diffraction peaks (Figure S2) [32]. In Figure S3, the peaks at 1617 and 3414 cm^−1^ in the FTIR spectrum of the Fe_3_O_4_@C confirm the presence of hydrophilic carboxyl groups on the surface of Fe_3_O_4_@C, which may be attributed to the oxidation of carbon facilitated by hydrogen peroxide [33]. The negative charge on the carboxyl groups at the surface of the shell layer provides electrostatic repulsion between the nanoparticles, enhancing stability in dispersed media. When an external magnetic field was applied to high-viscosity Fe_3_O_4_@C/EG solution, the magnetic nanoparticles were driven to align along the direction of magnetic field, forming one-dimensional chain structures and generating a photonic bandgap. Consequently, light at a specific wavelength is reflected. As the magnetic field strength increases, the photonic bandgap blue-shifts due to decreased spacing between the adjacent Fe_3_O_4_@C nanoparticles. In this process, the structural color gradually changes from red to blue, while the reflected peaks gradually shifted from 617 nm to 440 nm (Figure 1d,e).

In order to obtain structural colored OE-skins with tea polyphenols/borax, the initiator and monomer were added to Fe_3_O_4_@C/EG solution in order to form the pre-polymerization solution. The catechol groups of tea polyphenols in the pre-polymerization solution can form dynamic boronate ester bonds with boronate acid ions. Under a magnetic field, Fe_3_O_4_@C nanoparticles self-assemble into highly ordered one-dimensional chain-like structures in the pre-polymerization solution, and the interparticle distance can be adjusted by the intensity of the magnetic field. Finally, under the in situ UV polymerization, the aligned Fe_3_O_4_@C nanoparticles were immobilized, leading to the successful preparation of OE-skin with distinct reflective light properties (Figure 1f–h). As shown in the SEM image of TPC OE-skin (Figure S4), the Fe_3_O_4_@C nanoparticles self-assembled into one-dimensional chain-like structures, aligned along the direction of the external magnetic field.

**Figure 2:**
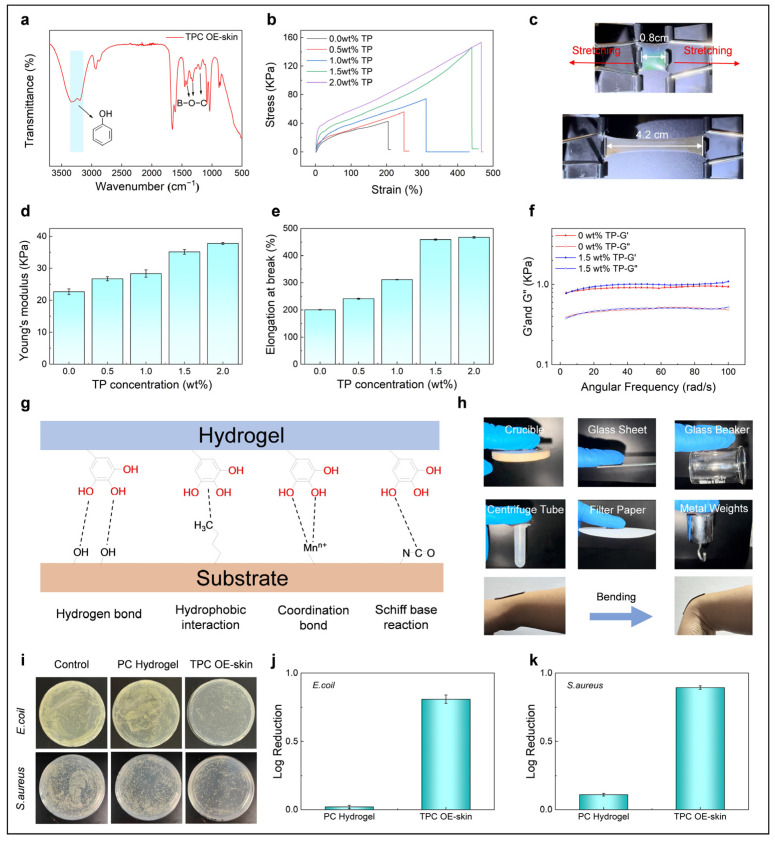
(**a**) FTIR spectrum of OE-skin; (**b**–**e**) Stress–strain curves, Young’s modulus, and elongation at break of OE-skin with varying TP contents; (**c**) Digital images of OE-skin before and during stretching; (**f**) Storage modulus (G′) and loss modulus (G′′) of OE-skin under angular frequency scanning; (**g**) Schematic illustration of the adhesion mechanism of OE-skin; (**h**) Digital photographs demonstrating the adhesion behavior of OE-skin on diverse surfaces; (**i**) Images of surviving bacterial colonies (*E. coli* and *S. aureus*) on culture plates after contact with PBS (Control), hydrogel without TP (PC hydrogel), and TPC OE-skin; (**j**,**k**) Antibacterial efficacy against *E. coli* and *S. aureus*.

In FTIR analysis, the broad reflection peak between 3400 and 3100 cm^−1^ can be attributed to the −OH stretching vibrations of TP, while the peaks at 1417 cm^−1^, 1324 cm^−1^, and 1202 cm^−1^ are indicative of the B−O−C bonds [34]. These results confirm the formation of dynamic boronate ester bonds between TP and borax (Figure 2a). PC hydrogels without TP or borax and TPC OE-skins incorporating four different concentrations of TP were prepared to examine the influence of TP concentration on mechanical properties (while fixing the weight ratio of TP to borax at 4:1). As shown in Figure 2b, all TPC OE-skins with added TP exhibited superior mechanical properties compared to the PC hydrogel. Additionally, as the concentration of TP increased, both the stress and strain of the TPC OE-skins progressively enhanced. Notably, the TPC OE-skin containing 1.5 wt.% TP exhibited remarkable stretching, from its original length of 0.8 cm to 4.2 cm without rupture (Figure 2c). Figure 2d presents the Young’s modulus of PC hydrogel and TPC OE-skins with varying amounts of TP. As the concentration of TP increases from 0 wt.% to 2.0 wt.%, the Young’s modulus of TPC OE-skin increases from 26 kPa to 44 kPa. The elongation at break trend aligns closely with the Young’s modulus, both increasing as the TP concentration rises (Figure 2e). The elongation at break of TPC OE-skin increases from 200% to 467%. Rheological measurements showed that the storage modulus (G’) of OE-skin exceeded the loss modulus (G″) over the frequency range of 0–100 rad/s, indicating viscoelastic solid behavior. The formation of TP-B slightly increased G’ but had minimal effect on G″, suggesting an improvement in viscoelasticity (Figure 2f). Moreover, the formation of TP-B significantly enhanced the conductivity of OE-skin (Figure S5). The adhesion performance of OE-skin is crucial for its application in human signal detection, ensuring seamless and reliable attachment to human skin. TP can form a variety of dynamic interactions, including hydrogen bonds, hydrophobic interactions, ion coordination, and Schiff base reactions [34,35], with various functional groups and ions. These interactions endow the OE-skin’s robust adhesion to both organic and inorganic surfaces (Figure 2g), such as ceramics, glass, polypropylene (PP), paper, and metal (Figure 2h). Interestingly, when the OE-skin is immersed in various solvents, such as water, methanol, acetone, ethylene glycol, and ethanol, it exhibits pronounced chromatic differences (Figure S6). These changes are attributed to variations in interparticle spacing caused by differences in swelling degrees, as well as the distinct refractive indices of the solvents, according to the Bragg’s law. This distinct solvent-responsive behavior enables the photonic crystal E-skin to achieve remarkable solvent recognition performance; thus, this may lay a solid foundation for the development of advanced environment-sensitive wearable devices.

To verify the biosafety of OE-skin, L-929 cells were utilized as model cells to evaluate the in vitro biocompatibility of the samples. The results demonstrated that the cell viability for both PC hydrogel and TPC OE-skin samples exceeded 99% (Figure S7), indicating that the incorporation of TP-B does not compromise the biocompatibility of OE-skin. The antibacterial properties of OE-skin were assessed by examining its effects on two common bacterial strains, *E. coli* and *S. aureus* (Figure 2i). The weak alkalinity of borax added to OE-skin creates an environment less conducive to bacterial proliferation. Furthermore, tea polyphenols, a class of plant-derived polyphenols, exhibit antibacterial activity by interfering with cellular metabolism and compromising cell membrane integrity [36]. To quantitatively assess antibacterial efficacy, the logarithmic reduction of bacterial counts was calculated. As illustrated in Figure 2j,k, the log reduction values for *E. coli* and *S. aureus* were 0.810 and 0.895, respectively. These findings confirm that the OE-skin group effectively inhibits the growth of bacteria, showcasing its notable antibacterial performance.

### 3.2. Optical and Electrical Sensing Performance of the TPC OE-Skin

**Figure 3:**
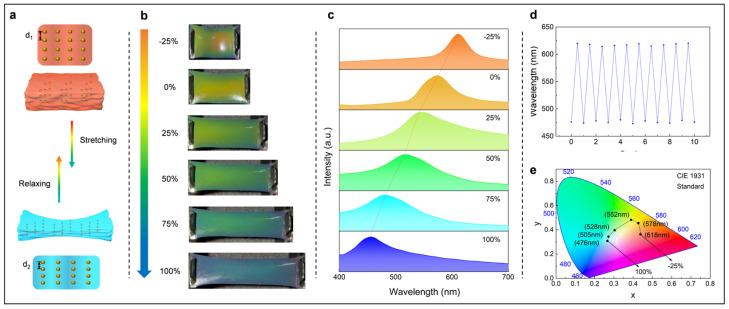
(**a**) Schematic illustration of the stretching and color-adjusting mechanism in the TPC OE-skin; (**b**,**c**) Images and corresponding reflectance spectra of the TPC OE-skin under strains ranging from −25% to 100%; (**d**) Reflection peak positions over 10 cycles of stretching and compression; (**e**) Chromaticity diagram illustrating the structural color changes under strains ranging from −25% to 100%.

Due to the excellent mobility of ions and the hydrogen bonding interactions between TP-B and the hydrogel polymer chains, the TPC OE-skin can deform in response to the stretching. Additionally, the ordered arrangement of Fe_3_O_4_@C nanoparticles within the hydrogel matrix endows the TPC OE-skin with excellent structural color. The wavelength of the structural color can be explained by the Bragg equation [37].

(5) $$\sqrt{\frac{8}{3}}D{\left(n_{eff}^{2}-{sin\theta }^{2}\right)}^{\frac{1}{2}}=m\lambda$$

where *D* is the lattice spacing, *n_eff_* is the effective refractive index, *θ* is the incident angle, *m* is the order of reflection, and *λ* is the wavelength of the reflected light. Among these parameters, the effective refractive and incident angle remain constant; thus, the observed structural color can be adjusted by varying the lattice spacing (Figure 3a). The TPC OE-skin was initially compressed by 25%, resulting in a visible change of structural color from orange to red. Upon further stretching the TPC OE-skin from 0 to 100%, the structural color transitioned from orange to blue (Figure 3b). Throughout this process, the reflected peaks gradually shifted from 618 nm to 476 nm (Figure 3c). Furthermore, TPC OE-skin showed slight changes in reflected peaks over 10 cycles of stretch and compression, indicating the stability of its mechanical discoloration (Figure 3d). The CIE color plot shows a wide range of color changes consistent with the experimental results (Figure 3e).

**Figure 4:**
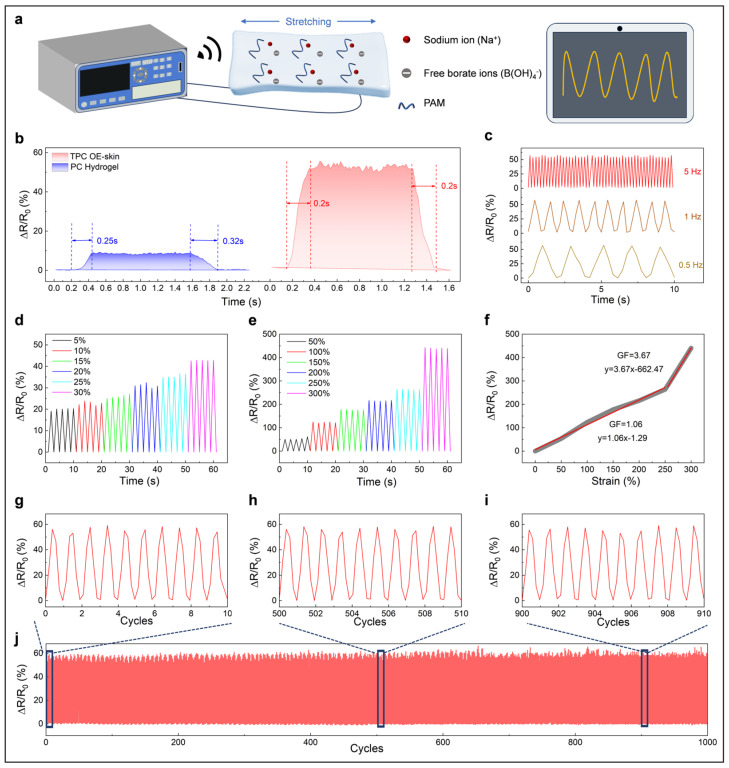
(**a**) Schematic illustration of the electrical signal detection process; (**b**) Analysis of response and recovery times for the OE-skin and PC hydrogel under a 50% strain condition; (**c**) Relative resistance changes of the OE-skin in response to 50% strain at frequencies of 0.5 Hz, 1 Hz, and 5 Hz; (**d**) Relative resistance changes under strains ranging from 5% to 30%; (**e**) Relative resistance changes under strains ranging from 50% to 300%; (**f**) The relative resistance–strain curve of the OE-skin with calculated GFs; (**g**–**j**) Relative resistance changes of the OE-skin during 1000 cycles at 50% strain.

The electrical sensing performance of TPC OE-skin was evaluated using a digital resistivity meter, with the TPC OE-skin clamped onto a reciprocating machine for controlled testing. The cross-sectional change of the E-skin under varying strain affects the ion transport speed, thereby altering its resistance (Figure 4a). Due to the presence of free borate ions and sodium ions in the OE-skin, the relative resistance change of the E-skin exhibits faster response/recovery time (200 ms) than that of the PC hydrogel without TP-B (250 ms in response and 320 ms in recovery) (Figure 4b). Additionally, in Figure 4c, the results of the relative resistance changes of the OE-skin under the same strain at varying frequencies demonstrate consistent and stable electrical signal transmission. In addition, as shown in Figure 4d,e, the OE-skin is capable of providing stable and distinguishable signals across small strains (5–30%) and large strains (50–300%). Gauge factor (GF) was detected to investigate the sensitivity of the OE-skin. As illustrated in Figure 4f, the OE-skin demonstrated two distinct linear response regions: a GF of 1.06 for strains ranging from 0% to 250%, and 3.67 for strains ranging from 250% to 300%. The enhanced sensitivity may potentially arise from the diminution in cross-sectional area, thereby elevating the resistance per unit length and amplifying the responsiveness. The OE-skin was further subjected to repeated stretching (50%) and releasing. In these cyclic tests, the relative resistance changes exhibited stable signal output over 1000 cycles, indicating the suitability of the OE-skin for long-term use. The exceptional optical and electrical performance of the TPC OE-skin underscores its strong potential for wearable applications demanding dual-signal response capabilities. Furthermore, the OE-skin demonstrates superior sensing performance and multifunctional properties, particularly in terms of tensile strain, electrical response time, adhesion, biocompatibility, and antibacterial characteristics, when compared with previously reported systems, as detailed in Table S2.

### 3.3. Wearable Sensor for Human Motion Detection

**Figure 5:**
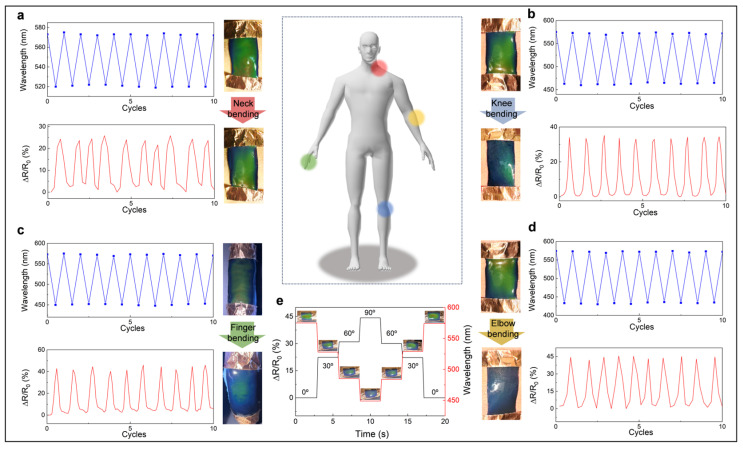
Demonstration of the synergistic electronic/optical dual-mode sensing in motion detection using the TPC OE-skin for (**a**) neck, (**b**) knee, (**c**) finger, and (**d**) elbow. (**e**) The reflection wavelengths and relative resistance changes of TPC OE-skin at various finger bending angles.

Based on its excellent mechanochromic properties, high sensitivity, and stable performance in electrical sensing, the TPC OE-skin can be functionalized as a wearable and flexible sensing device for monitoring human movements with high precision and reliability. A digital electric bridge was employed to record the electrical signals of the OE-skin, while the wavelength was measured using a fiber optic spectrometer. During motion monitoring, the color changes of the OE-skin can be directly observed with the naked eye. As shown in Figure 5a–d, due to the ability to synchronously output optical and electrical signals of the OE-skin, the bending of the joint can be easily recognized by both the resistance and structural color (neck, elbow, finger, knee). In the case of neck bending, the bending-induced stretching of the OE-skin can trigger synchronous changes in resistance and structural color instantaneously. Specifically, while the neck bend is 30°, 25% changes in relative resistance can be detected, and the structural color changed from yellow–green to green, with corresponding reflection wavelength changed from 575 nm to 525 nm. The 10 cyclic tests show that cyclic neck bending and head raising can be detected via reversed stable optical and electrical signals, enabling precise motion tracking. Additionally, stable synergistic electronic and optical sensing for elbow, finger, and knee motions are demonstrated. In addition, as shown in Figure 5e, during a single cycle of finger bending and releasing, the electrical signals and optical signals at bending angles of 0°, 30°, 60°, and 90° exhibit simultaneous and consistent variations, providing stronger evidence for the synchronization of dual-mode sensing. In Figure S8, we notice that the TPC OE-skin undergoes structural color changes after immersion in a saline solution, suggesting its unsuitability for operation under sweaty conditions. However, the long-term use tests, conducted by monitoring finger motions, demonstrate that the TPC OE-skin can maintain its performance in the real world in both optical and electrical signals over 200 cycles with minimal fluctuation (Figure S9).

## 4. Conclusions

In conclusion, we have successfully developed a photonic crystal hydrogel-based TPC OE-skin that is capable of delivering synergistic electrical and optical signals for the precise detection of joint motions. The TPC OE-skin was fabricated by integrating tea polyphenols and borax into a polyacrylamide hydrogel containing aligned Fe_3_O_4_@C nanoparticles. Upon deformation, the OE-skin exhibits structural color variation through the regulation of lattice spacing. Moreover, the incorporation of tea polyphenols endows the OE-skin with excellent antibacterial activity and biocompatibility. The formation of dynamic boronate ester bonds between tea polyphenols and borax also significantly enhances the mechanical strength and electrical conductivity of the OE-skin. Importantly, the OE-skin demonstrates rapid response/recovery times (200 ms) and high sensitivity (GF = 1.06 at strains from 0% to 250%, and 3.67 at strains from 250% to 300%) in electrical signal transmission based on resistance changes. Additionally, the OE-skin provides stable signal output during long-term use. Furthermore, the developed TPC OE-skin effectively showcases its capability for synergistic electronic and optical sensing during human motion monitoring for various joints, highlighting its broad application potential in the field of smart wearable sensors.

## Data Availability

Data are contained within the article.

## Supplementary Materials

The following supporting information can be downloaded at: https://www.mdpi.com/article/10.3390/bios15050281/s1, Figure S1: Hysteresis loops of the Fe_3_O_4_@C nanoparticles; Figure S2: XRD pattern of the Fe_3_O_4_@C nanoparticles; Figure S3: FT-IR spectra of Fe_3_O_4_@C; Figure S4: SEM image of aligned Fe_3_O_4_@C nanoparticles within the TPC OE-skin; Figure S5: Conductivity of TPC OE-skin with different TP contents; Figure S6: The solvent-response behaviour of the TPC OE skin; Figure S7: The biocompatibility of the TPC OE-skin; Figure S8: The image and reflection spectra of the TPC OE-skin before and after immersion in saline solution; Figure S9: The reflection wavelength and relative resistance changes of OE skin during 200 cycling tests of finger bending; Table S1: The formulations of PC hydrogel and TPC OE-skins with different content of TP; Table S2: Comparison of the sensing performance and functionalities between our TPC OE-skin and other reported dual-signal sensors.
