# Supplementary material for: Optical–Electronic Skin Based on Tea Polyphenol for Dual Signal Wearable Sensing
Source: Biosensors (Basel). 2025 Apr 29;15(5):281. doi: 10.3390/bios15050281 (PMC12110438; doi:10.3390/bios15050281)
Supplement: Supplementary file 1 [file biosensors-15-00281-s001.zip › biosensors-3579602-supplementary.pdf]

Supplementary Materials

Optical-Electronic Skin Based on Tea Polyphenol for Dual Signal Wearable Sensing

Jia-Li Xu [1,†], Guangyao Zhao [2,†], Jiachen Wang [2], An Tang [1], Jun-Tao Liu [1], Zhijie Zhu [3], Qiang Zhang [2,4,*] and Yu Tian [1,*]

[1] School of Chemistry and Chemical Engineering, Key Laboratory of Surface & Interface Science of Polymer Materials of Zhejiang Province, Zhejiang Sci-Tech University, Hangzhou 310018, China;
[2] Department of Biomedical Engineering, City University of Hong Kong, Kowloon, Hong Kong 999077, China;
[3] Jiangsu Advanced Textile Engineering Technology Center, Jiangsu College of Engineering and Technology, Nantong 226007, China;
[4] Institute of Digital Medicine, City University of Hong Kong, Kowloon, Hong Kong 999077, China
* Correspondence: qiang.johnny.zhang@cityu.edu.hk (Q.Z.); tianyu_zstu@zstu.edu.cn (Y.T.)
† These authors contributed equally to this work.

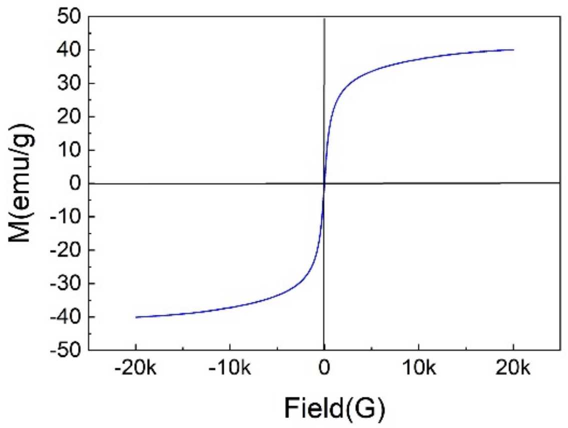

Figure S1. Hysteresis loops of the Fe_3O_4@C nanoparticles.

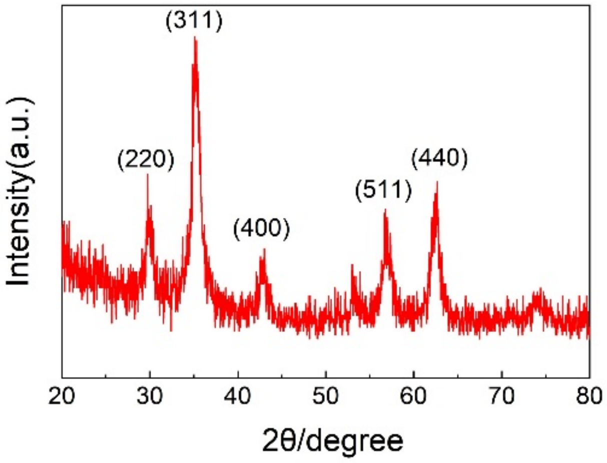

Figure S2. XRD pattern of the Fe_3O_4@C nanoparticles.

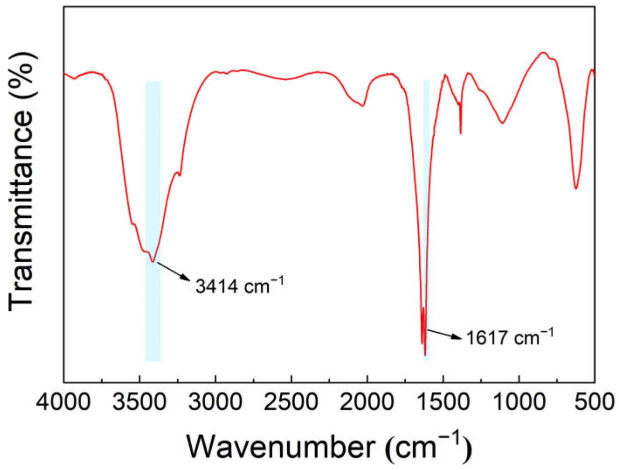

Figure S3. FT-IR spectra of Fe_3O_4@C.

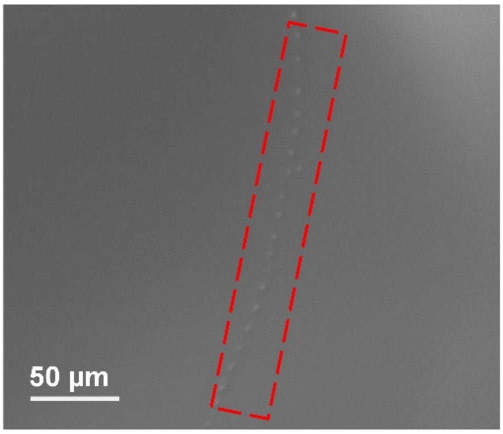

Figure S4. SEM image of aligned Fe_3O_4@C nanoparticles within the TPC OE-skin.

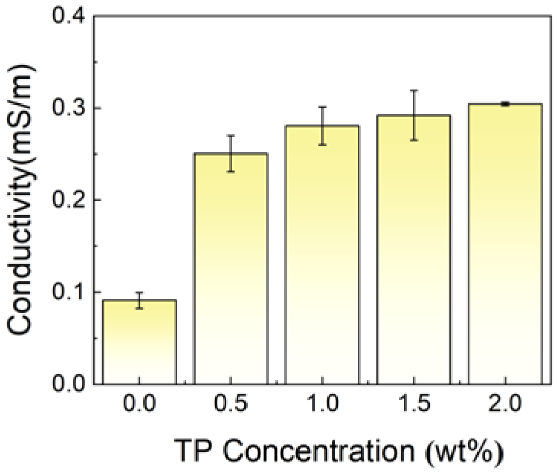

Figure S5. Conductivity of TPC OE-skin with different TP contents.

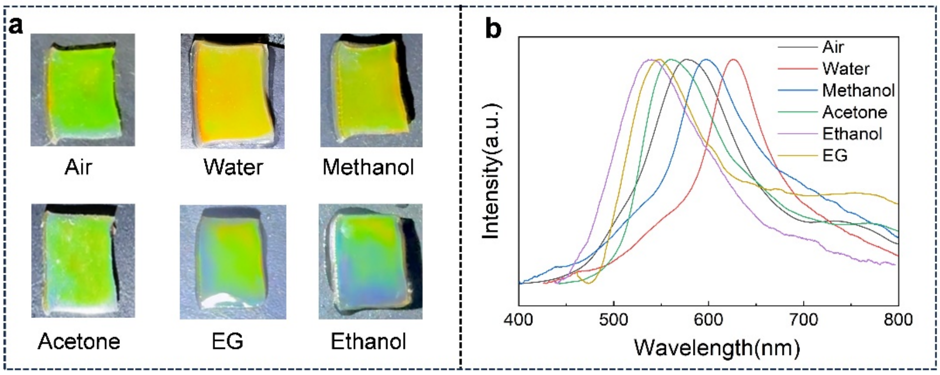

Figure S6. The solvent-response behaviour of the TPC OE skin. (**a**) Digital photos of TPC OE-skin immersed in different solvents; (**b**) Reflectance spectra of TPC OE-skin immersed in different solvents.

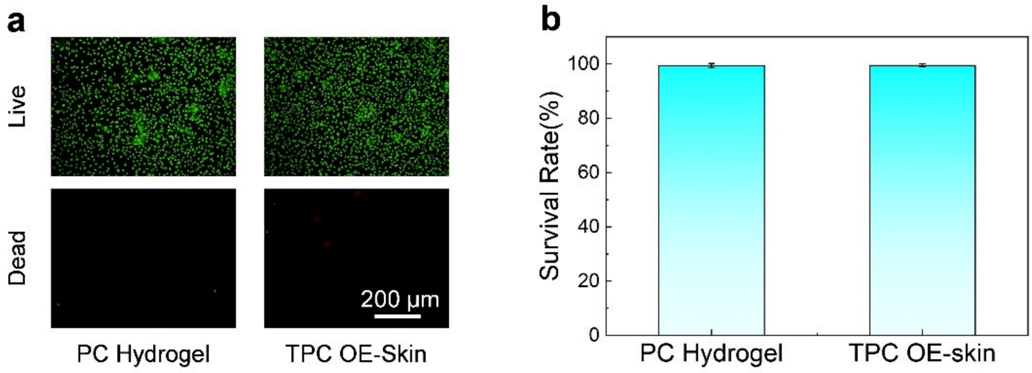

Figure S7. The biocompatibility of the TPC OE-skin. (**a**) Live/Dead staining of L-929 cells after treated with PC hydrogel and TPC OE-skin for 24 hours. Scale bar: 200 μm; (**b**) The L-929 cells survival rate of PC hydrogel and TPC OE-skin.

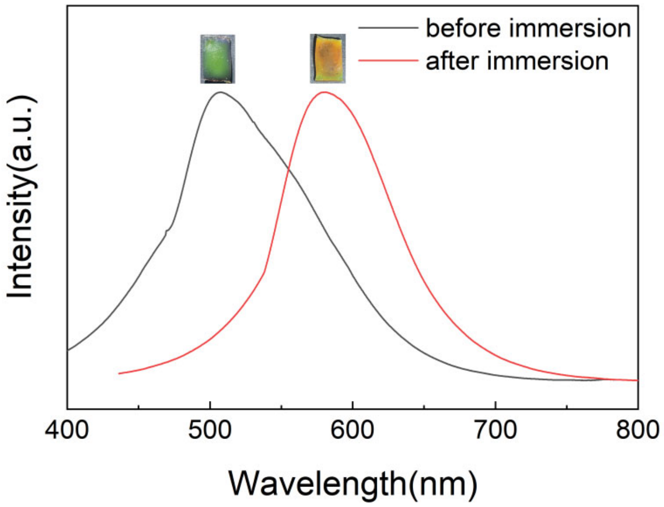

Figure S8. The image and reflection spectra of the TPC OE-skin before and after immersion in saline solution.

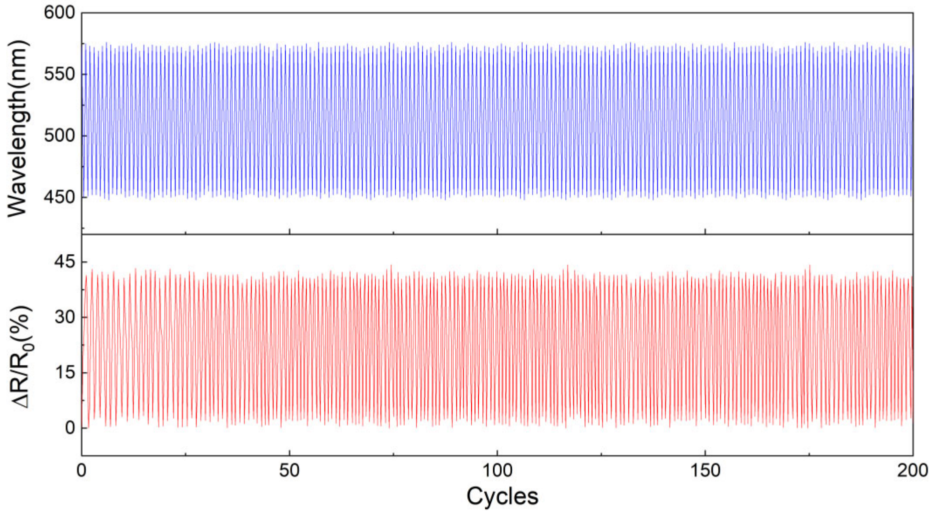

Figure S9. The reflection wavelength and relative resistance changes of OE skin during 200 cycling tests of finger bending.

Table S1. The formulations of PC hydrogel and TPC OE-skins with different content of TP.

**Sample**	**The Content of TP**	**TP(mg)**	**Borax(mg)**	**Am(g)**	**Fe_3O_4@C/EG(mL)**	**EG(mL)**
PC	0%	0	0	1.6	1	1.4
TPC-0.5%	0.5%	20	5	1.6	1	1.4
TPC-1.0%	1.0%	40	10	1.6	1	1.4
TPC-1.5%	1.5%	60	15	1.6	1	1.4
TPC-2.0%	2.0%	80	20	1.6	1	1.4

Table S2 Comparison of the sensing performance and functionalities between our TPC OE-skin and other reported dual-signal sensors.

**Dual-signal sensors**		**Matrix**	**Mechanism**	**Strain**	**Electrical response time**	**Adhesion**	**Biocompatibility**	**Anti-bacterial**	**Observe method**	**Ref.**
**Optical**	**Electrical**									
Cellulose nanocrystal	CNTs	Polyacrylamide	Structural color	Over 250%	N	Y	Y	N	Naked Eyes	1
$W_{18}O_{49}$ nanowires and Au nanoparticles@polyaniline	Flexible conductive substrate	PDMS	Electrochemiluminescence	N	<3s	Y	N	N	Electrical stimulus	2
Swollen self-assembled CNC film with helical nanoarchitectures	Fluorine-richionic liquids	Poly(2-hydroxyethyl acrylate)	Structural color	Over 200%	500 ms	N	Y	N	Naked Eyes	3
PS@SiO_2 photonic arrays	MXene	Poly(ethylene glycol methyl ether acrylate)	Structural color	0-70%	N	N	N	N	Naked Eyes	4
ZnS@SiO_2 photonic arrays	Free ions (Na^+, and Cl^-)	Polyacrylamide	Structural color	Around 400%	N	N	N	N	Naked Eyes	5
Tris(2,2′-bipyridine) ruthenium (II) hexafluorophosphate([Ru(bpy)$_3$][PF$_6$]$_2$	1-ethyl-3-methylimidazolium bis(trifluoromethanesulfonyl) imide	Polyurethane	Electrochemiluminescence	0-100%	N	N	N	N	Pressure applied	6
Non-close packed array of SiO_2	MXene	Polyurethane	Structural color	0-191.8 %	N	Y	Y	N	Naked Eyes	7
Inverse opal structure of SiO_2	CNT	polyacrylamide - gelatin	Structural color	over 250%	N	Y	Y	N	Naked Eyes	8
Red fluorescent RB-PHEAA	CNTs	PDMS	Mechanoflourensce	0-100%	N	N	N	N	UV Light	9

Wurtzite-structure of ZnS microparticles	Polyaniline @cellulose nanocrystals	PDMS	Mechanofl uorensce	0-70%	120 ms	N	N	N	UV Light	10
Aligned magnetic Fe_3O_4@C	Tea polyphenol and borax	Polyacryla mide	Structural color	458.9 %	200 ms	Y	Y	Y	Naked Eyes	**This wor k**

References

1. Wang, X.; Geng, M.; Pan, X.; Wang, Y.; Zhan, T.; Liu, Y.; Li, J.; Ma, X.; Zhang, Z.; Gao, M. Skin-Adherent, Cellulose-Based Photonic Patch for Visual Strain Mapping. *ACS Mater. Lett.* **2025,** *7*, 854-861. http://doi.org/10.1021/acsmaterialslett.4c01845.
2. Yu, Y.; Zhu, X.; Jiang, S.; Wu, S.; Zhao, Y.; Zhang, L.; Song, L.; Huang, Y. Cephalopods' Skin-Inspired Design of Nanoscale Electronic Transport Layers for Adaptive Electrochromic Tuning. *Adv. Sci.* **2024,** *11*, 2405444. http://doi.org/10.1002/advs.202405444.
3. Li, X.; Yang, Y.; Valenzuela, C.; Zhang, X.; Xue, P.; Liu, Y.; Liu, C.; Wang, L. Mechanochromic and Conductive Chiral Nematic Nanostructured Film for Bioinspired Ionic Skins. *ACS Nano* **2023**, *17*, 12829–12841. http://doi.org/10.1021/acsnano.3c04199.
4. Li, S.; Wang, J.; Xiao, Y.; Dai, P.; Wang, Y.; Zhang, H.; Shan, G.; Jia, L. Chameleon-inspired structural color fabrics with photoelectric dual-signal outputs for human motion monitoring. *J. Colloid Interface Sci.* **2025,** *692*, 137538. http://doi.org/10.1016/j.jcis.2025.137538.
5. Xu, J.; Li, Y.; Yao, Y.; Ding, Y.; Tan, Y.; Hu, G.; Zhang, S.; Zeng, L. A Stimulus-Responsive Optoelectronic Skin From Photonic Crystal. *Adv. Opt. Mater.* **2025**, 2500094. http://doi.org/10.1002/adom.202500094.
6. Lee, J. I.; Choi, H.; Kong, S. H.; Park, S.; Park, D.; Kim, J. S.; Kwon, S. H.; Kim, J.; Choi, S. H.; Lee, S. G.; Kim, D. H.; Kang, M. S. Visco-Poroelastic Electrochemiluminescence Skin with Piezo-Ionic Effect. *Adv. Mater.* **2021,** *33*, 2100321. http://doi.org/10.1002/adma.202100321.
7. Liu, C.; Zhao, Q.; Cao, Y.; Li, X.; Peng, K.; Fu, F., Bioinspired Structural Color Hydrogel Skin from Nonclose-Packed Colloidal Crystal Arrays for Epidermal Sensing. *ACS Appl. Mater. Interfaces* **2025,** *17*, 16658-16667. http://doi.org/10.1021/acsami.5c01011.
8. Li, W.; Li, J.; Ding, X.; Tan, Q.; Sun, W.; Lai, P.; Zhao, Y. Multi-bioinspired electronic skins with on-demand adhesion and opto-electronic synergistic display capabilities. *The Innovation* **2025**, 100877. http://doi.org/10.1016/j.xinn.2025.100877.
9. Lin, G.; Si, M.; Wang, L.; Wei, S.; Lu, W.; Liu, H.; Zhang, Y.; Li, D.; Chen, T. Dual-Channel Flexible Strain Sensors Based on Mechanofluorescent and Conductive Hydrogel Laminates. *Adv. Opt. Mater.* **2022,** *10*, 2102306. http://doi.org/10.1002/adom.202102306.
10. Yang, X.; Zhang, X.; Guan, Q.; Zhang, X. Biomimetic multifunctional E-skins integrated with mechanoluminescence and chemical sensing abilities. *J. Mater. Chem. C* **2021,** *9*, 2815-2822. http://doi.org/10.1039/d0tc05499b.
